# Viral and Bacterial Profiles in Endemic Influenza A Virus Infected Swine Herds Using Nanopore Metagenomic Sequencing on Tracheobronchial Swabs

**DOI:** 10.1128/spectrum.00098-23

**Published:** 2023-02-28

**Authors:** Nick Vereecke, Sophia Zwickl, Sophie Gumbert, Annika Graaf, Timm Harder, Mathias Ritzmann, Kathrin Lillie-Jaschniski, Sebastiaan Theuns, Julia Stadler

**Affiliations:** a Laboratory of Virology, Faculty of Veterinary Medicine, Ghent University, Merelbeke, Belgium; b PathoSense BV, Lier, Belgium; c Clinic for Swine at the Centre for Clinical Veterinary Medicine, LMU Munich, Germany; d Institute of Diagnostic Virology, Friedrich-Loeffler-Institut, Germany; e CEVA Tiergesundheit, Germany; Texas A&M University

**Keywords:** domestic pigs, diagnostics, nanopore sequencing, influenza A virus, coinfections

## Abstract

Swine influenza A virus (swIAV) plays an important role in porcine respiratory infections. In addition to its ability to cause severe disease by itself, it is important in the multietiological porcine respiratory disease complex. Still, to date, no comprehensive diagnostics with which to study polymicrobial infections in detail have been offered. Hence, veterinary practitioners rely on monospecific and costly diagnostics, such as Reverse Transcription quantitative PCR (RT-qPCR), antigen detection, and serology. This prevents the proper understanding of the entire disease context, thereby hampering effective preventive and therapeutic actions. A new, nanopore-based, metagenomic diagnostic platform was applied to study viral and bacterial profiles across 4 age groups on 25 endemic swIAV-infected German farms with respiratory distress in the nursery. Farms were screened for swIAV using RT-qPCR on nasal and tracheobronchial swabs (TBS). TBS samples were pooled per age, prior to metagenomic characterization. The resulting data showed a correlation between the swIAV loads and the normalized reads, supporting a (semi-)quantitative interpretation of the metagenomic data. Interestingly, an in-depth characterization using beta diversity and PERMANOVA analyses allowed for the observation of an age-dependent interplay of known microbial agents. Also, lesser-known microbes, such as porcine polyoma, parainfluenza, and hemagglutinating encephalomyelitis viruses, were observed. Analyses of swIAV incidence and clinical signs showed differing microbial communities, highlighting age-specific observations of various microbes in porcine respiratory disease. In conclusion, nanopore metagenomics were shown to enable a panoramic view on viral and bacterial profiles as well as putative pathogen dynamics in endemic swIAV-infected herds. The results also highlighted the need for better insights into lesser studied agents that are potentially associated with porcine respiratory disease.

**IMPORTANCE** To date, no comprehensive diagnostics for the study of polymicrobial infections that are associated with porcine respiratory disease have been offered. This precludes the proper understanding of the entire disease landscape, thereby hampering effective preventive and therapeutic actions. Compared to the often-costly diagnostic procedures that are applied for the diagnostics of porcine respiratory disease nowadays, a third-generation nanopore sequencing diagnostics workflow presents a cost-efficient and informative tool. This approach offers a panoramic view of microbial agents and contributes to the in-depth observation and characterization of viral and bacterial profiles within the respiratory disease context. While these data allow for the study of age-associated, swIAV-associated, and clinical symptom-associated observations, it also suggests that more effort should be put toward the investigation of coinfections and lesser-known pathogens (e.g., PHEV and PPIV), along with their potential roles in porcine respiratory disease. Overall, this approach will allow veterinary practitioners to tailor treatment and/or management changes on farms in a quicker, more complete, and cost-efficient way.

## INTRODUCTION

Respiratory disease is a major issue compromising animal health, economic success, and welfare in the swine industry. In many cases, the clinical outcome is a result of a complex interplay of viral and bacterial pathogens, which is often referred to as the porcine respiratory disease complex (PRDC). This describes a clinical condition that often manifests as a treatment-resistant respiratory disease in the nursery and in growing pigs of multifactorial etiology, including infectious and noninfectious factors (1–4). Swine influenza A virus (swIAV) is considered to be an important primary respiratory pathogen, and it also possesses a zoonotic propensity. Without complication, an acute swIAV infection recedes quickly, and viral shedding stops within seven days after the onset of an infection (5). Hence, diagnosing swIAV virologically requires sampling within this short infectious period of five to seven days. Even though the virus itself can be seen as a predisposing factor, its clinical outcome is highly dependent on the swIAV subtype and associated pathogens. As swIAV can act as a promoter for other primary and secondary pathogens, concurrent and successive infections frequently occur (6–8). The polymicrobial nature of porcine respiratory diseases, with the often nonsimultaneous (i.e., consecutive) occurrence of the different pathogens, complicates effective diagnostics and tailored interventions (9). The interactions between swIAV and viral or bacterial coinfections have been extensively studied *in vitro*, *ex vivo*, and *in vivo*, and this work has been extensively reviewed by Saade and colleagues (2020) (10). Unfortunately, most of the reviewed experiments focused on the immunological response or clinical outcome, and barely any of these trials addressed the disease outcome from the perspective of infection dynamics. In addition, mainly dual infection trials were conducted, but these do not allow for the study of the complex interplay of swIAV and multiple coinfecting microorganisms. The results of these experimental set-ups often cannot be extrapolated to the complex field situation, in which confounders, such as the environment and management, also impact the disease onset, propagation, and outcome. Therefore, the authors concluded that a significant amount of coinfection data are lacking and that many discrepancies between experiments exist (10). This is mainly due to the experimental set-up; the choice of viral/bacterial strains, route of inoculation, multiplicity of infection, pig breed, and/or farm health-status are all parameters that highly impact the final experimental outcome. To elucidate the dynamics of respiratory microorganisms, more extensive field studies are urgently required. This would also allow for the understanding of the roles of lesser studied (e.g., porcine hemagglutinating encephalomyelitis virus [PHEV]) or potentially new porcine respiratory pathogens (e.g., porcine parainfluenza virus [PPIV]) in disease outcome/progression. A recent study from Martin-Valls et al. (2022) aimed to investigate nasal swabs for 11 respiratory viruses in endemically swIAV-infected herds. However, bacterial agents causing respiratory disease were not included in the analysis. In addition, the study used nasal swabs, which are not favorable as detection material for viruses that do not replicate in the nasal mucosa, thereby posing another limitation (11–13).

Until recently, veterinarians were limited in their diagnostic options, although real-time or quantitative PCR, rapid antigenic tests, and antibody-dependent assays have allowed them to perform targeted diagnostics in a fast manner. Depending on which approach is applied, the costs associated with respiratory disease diagnostics range from relatively low (e.g., serology) to high (e.g., bacterial cultures, typing, and multiple PCRs) (14). As with these diagnostic tools, a prior selection of pathogens to be examined must be made, meaning that manifestations of polymicrobial infections might not be identified correctly. Furthermore, these methods often lack comprehensive knowledge of the complete etiology, which bears the risk of incorrect therapeutic (e.g., antimicrobial treatment) and preventive action plans (e.g., vaccination). Multiplex qPCR approaches may help to bundle polyetiological diagnostic approaches, and such assays have been designed for the bovine respiratory disease complex (BRDC), which has a similar polymicrobial etiology; however, they are often incomplete, are sensitive to primer mismatching due to viral evolution, and come with a higher cost (15). Comparable multiplexing alternatives in porcine respiratory disease and PRDC have been generated, but they are not widely used in routine diagnostic laboratories (16). With the increased availability of new sequencing technologies, the costs associated with sequencing could be significantly reduced. The release of third-generation sequencing methods, such as Oxford Nanopore Technologies’ (ONT) nanopore sequencing, represented a new era in which to study and diagnose infectious diseases. In this way, a broad overview of viruses and bacteria, as part of porcine respiratory diseases, can be explored. As exemplified for a wide variety of diseases in both human and veterinary medicine, targeted sequencing and metagenomic protocols allow for the study of infectious diseases in a quick and cost-efficient manner (17–23). Moreover, (semi-)quantitative statements regarding the relative abundancies of various infectious agents are possible from a mixed sample (22, 23). Looking at the total nucleic acid content of a diagnostics sample, also known as metagenomics, has been shown to be an interesting means by which to study microbial profiles within a sample, although most studies lack the combined observation (and interpretation) of viral and bacterial players within the porcine disease context (24, 25). These studies relied on short-read Illumina sequencing, although third-generation sequencing with *ad random* amplification of viral and bacterial agents might be an interesting solution by which to study polymicrobial infections. This methodology has recently become available not only to researchers but also to the field of veterinary practitioners (22, 23).

Hence, the aim of this study was to evaluate the use of third-generation, nanopore-based metagenomics in the identification of coinfections (both viral and bacterial) from tracheobronchial swabs (TBS) in endemic swIAV-infected farms.

## RESULTS

### Estimating swIAV incidence across studied farms.

**FIG 1:**
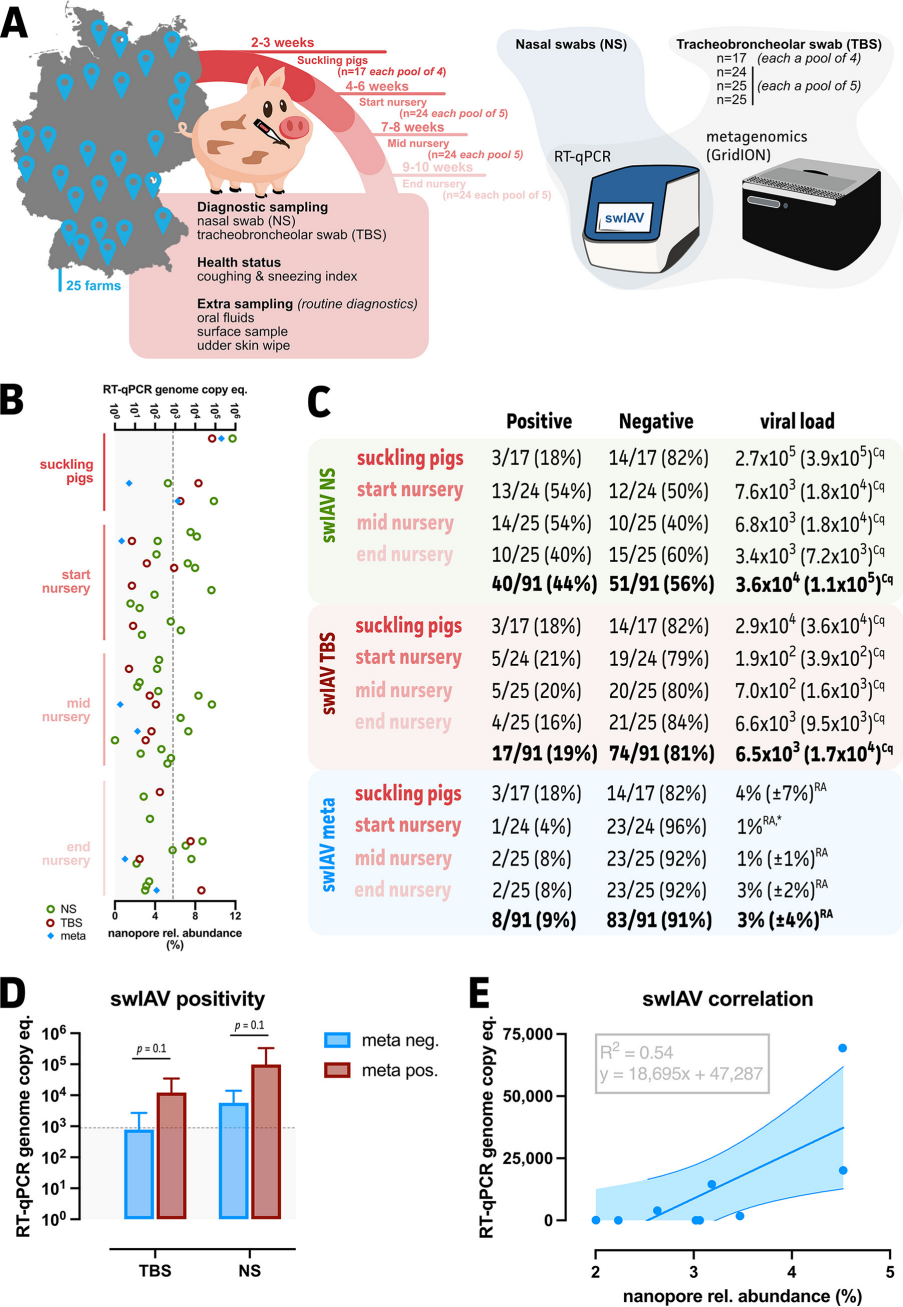
Estimation of swIAV incidence across 25 German farms. (A) Schematic overview of the experimental set-up, highlighting different sample availabilities and metadata. (B) Overview of swIAV viral loads (RT-qPCR) across different farms and age groups for nasal swabs (NS; green open circles), tracheobronchial swabs (TBS; red open circles), and metagenomics (meta; blue squares). (C) Tabular overview of swIAV occurrence (Cq, genome copy equivalents per 0.1 mL for NS and TBS samples, based on RT-qPCR viral loads; RA, relative abundances [%] for metagenomics, based on swIAV classified reads, with SD in parentheses) across age groups and different applied methods. An asterisk (*) indicates that an insufficient number of data points were available to determine the standard deviation (SD). (D) swIAV viral loads (RT-qPCR) for TBS and NS samples, in relation to metagenomics detection. A statistical analysis was performed using a multiple Wilcoxon test. (E) Simple linear regression was used to assess the correlation between swIAV RT-qPCR loads from TBS samples and nanopore relative abundances (%). The dotted lines represent the Cq 30 values (or 886 genome copy equivalents per 0.1 mL).

Overall, swIAV was detected in 17 out of the 25 included farms in nasal swabs via RT-qPCR. In the remaining farms, either swIAV was detected in other sampling materials (environmental samples or oral fluids, data not shown) or antibodies against swIAV were evident by a hemagglutination inhibition test (data not shown). In 3 out of the 17 nasal swab positive farms, swIAV was found in suckling piglets, and in 15 out of the 17 nasal swab positive farms, swIAV was found in weaners (Fig. 1B and C; Table S1). In total, 91 pooled TBS samples were obtained from the 25 German farms. These samples were subjected to both RT-qPCR and metagenomic sequencing to detect swIAV. Whereas the RT-qPCR analyses on the NS and TBS samples resulted in a total of 40 (44% of all samples) and 17 (19% of all samples) swIAV positive samples, respectively, nanopore metagenomics only showed 8 positive TBS samples (9% of all samples). An overview of the swIAV detection per farm and age group can be found in Fig. 1B and C and in Table S1. If a sample was found to be positive via nanopore metagenomics (*n* = 8), it was also found to be positive using RT-qPCR on TBS and the associated NS sample. Five samples showed swIAV detection in both the NS and TBS samples but not via metagenomics. From the 40 NS samples in which swIAV was identified with a mean viral load of 186 ± 175 genome copy equivalents per 0.1 mL, 25 showed no detection of swIAV in either TBS or metagenomics. Also, all age groups that were found to be swIAV positive for TBS samples (RT-qPCR) were positive in the nasal swabs, except for four samples. These samples showed low viral loads (25 ± 19 genome copy equivalents per 0.1 mL). TBS samples testing positive via nanopore metagenomics showed average genome copy equivalents of 778 ± 750 (per 0.1 mL), compared to 66 ± 60 for samples in which no swIAV was detected via this method. Even though a trend could be observed (i.e., lower genome copy equivalents for metagenomic samples negative for swIAV for both TBS and NS), no significant differences were found between the genome copy equivalents (RT-qPCR) of the nanopore metagenomics positive and negative TBS samples (Fig. 1D). The same conclusions could be drawn when looking at the NS RT-qPCR genome copy equivalents. The samples that were designated positive in both tests were used to address the (semi-)quantification of swIAV using normalized nanopore relative abundances. This showed a correlation with a lowered R squared value (0.51) (Fig. 1E).

### Detection of viral and bacterial organisms across German farms.

**FIG 2:**
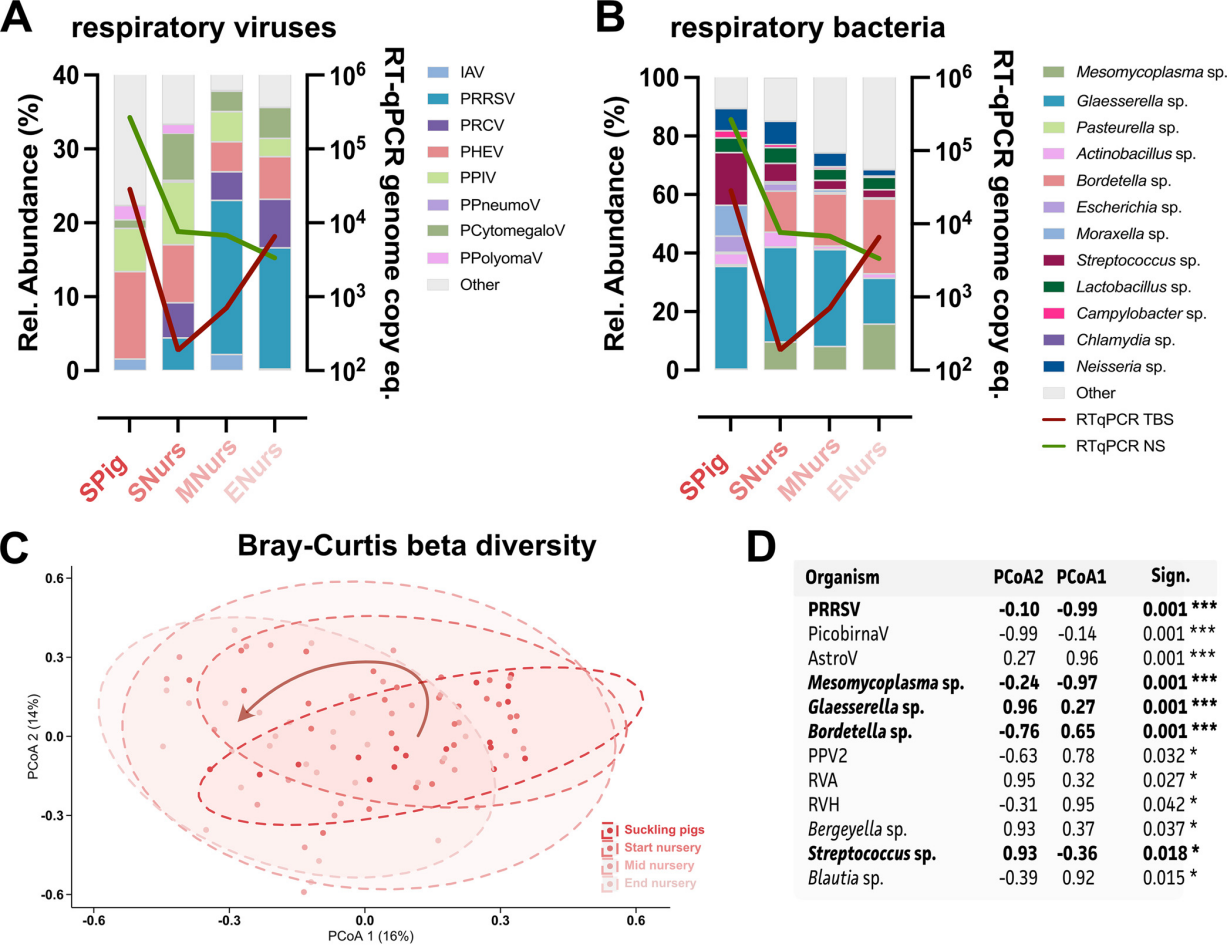
Age-related mean relative abundances of relevant respiratory viruses and bacteria in metagenomic analyses for the 25 swine farms. (A) Mean relative abundances (%) of relevant respiratory viruses across age groups. (B) Mean relative abundances (%) of relevant respiratory bacteria across age groups. Red and green lines represent RT-qPCR swIAV viral loads for TBS and NS samples, respectively, as represented by the genome copy equivalents per 0.1 mL. (C) Principal coordinate analysis (PCoA) of the four age groups for all microbial agents with PCoA 1 (*x*-axis) and PCoA 2 (*y*-axis) representing the highest diversity amongst our groups, the arrow suggests the evolution of the populations over time. (D) Results of statistical analysis using PERMANOVA, highlighting respiratory-associated pathogens and their factor loads per PCoA axis. Statistical significance is indicated by *, *P* < 0.05; **, *P* < 0.01; ***, *P* < 0.001.

Our study identified the distribution of viral and bacterial PRDC-related and nonrelated microbes from TBS samples across 25 farms that were suspected to have swIAV. As summarized in Table 1 and in Fig. 2A and B, PRRSV was identified in 14 out of the 25 farms (56%), and it was only detected from the nursery stage onwards. Next, PHEV and PPIV were also identified in nearly half of the farms (48% and 40%, respectively). Lower abundances were observed for porcine polyomavirus (PPolyomaV), atypical porcine pestivirus (APPV), PRCV, and porcine pneumovirus (PPneumoV). Interestingly, PRCV and PPneumoV were not detected in the TBS samples that were taken from piglets in the suckling stage. A 12.5% detection rate for PRRSV was seen at the beginning of the nursery, and this increased to up to 40% at the end of the nursery. Nearly all (96%) of the farms were positive for porcine cytomegalovirus (PCytomegaloV). Again, the virus was not detected in the TBS samples that were taken from suckling piglets, but it was highly abundant in all stages of the nursery. Also, astro- and picobirnaviruses were identified on nearly all of the farms (100% and 96%, respectively), showing a peak at the start of the nursery. However, they are not considered to be PRDC-associated viruses. The same accounted for the detection of entero- and rotaviruses (76% and 52%, respectively). For bacteria, the most abundant in circulation (>90%) were Glaesserella, Streptococcus, Lactobacillus, Mesomycoplasma, and Prevotella species. Whereas most of these species circulated at any of the age groups, only 23.5% of farms were positive for *Mesomycoplasma* species at the suckling piglet stage, compared to 72 to 83% throughout the nursery period. The next group of bacteria (present at 60 to 90% of all farms) was composed out of Neisseria, Bordetella, Faecalibacterium, Moraxella, Campylobacter, Rothia, and Corynebacterium species. Whereas the *Bordetella* species showed a steady increase from suckling piglets up to the end of nursery, a decline in *Moraxella* species was observed (Table 1; Table S1). Moreover, no *Faecalibacterium*-positive suckling piglets were identified, compared to 42 to 52% in the nursery period. Comparable to the decline in *Moraxella* species, reductions in *Actinobacillus* and *Bergeyella* species were also observed, and these were found on 56% and 48% of the farms, respectively. The less abundant bacteria were Parabulkholderia, Escherichia, Pasteurella, Chlamydia, Coprococcus, and Blautia species (<30%). Here, again, Chlamydia and *Coprococcus* species were not identified in suckling piglets.

**TABLE 1:** Detection of viral and bacterial agents in tracheobronchial samples of pigs from 25 German farms^*a*^

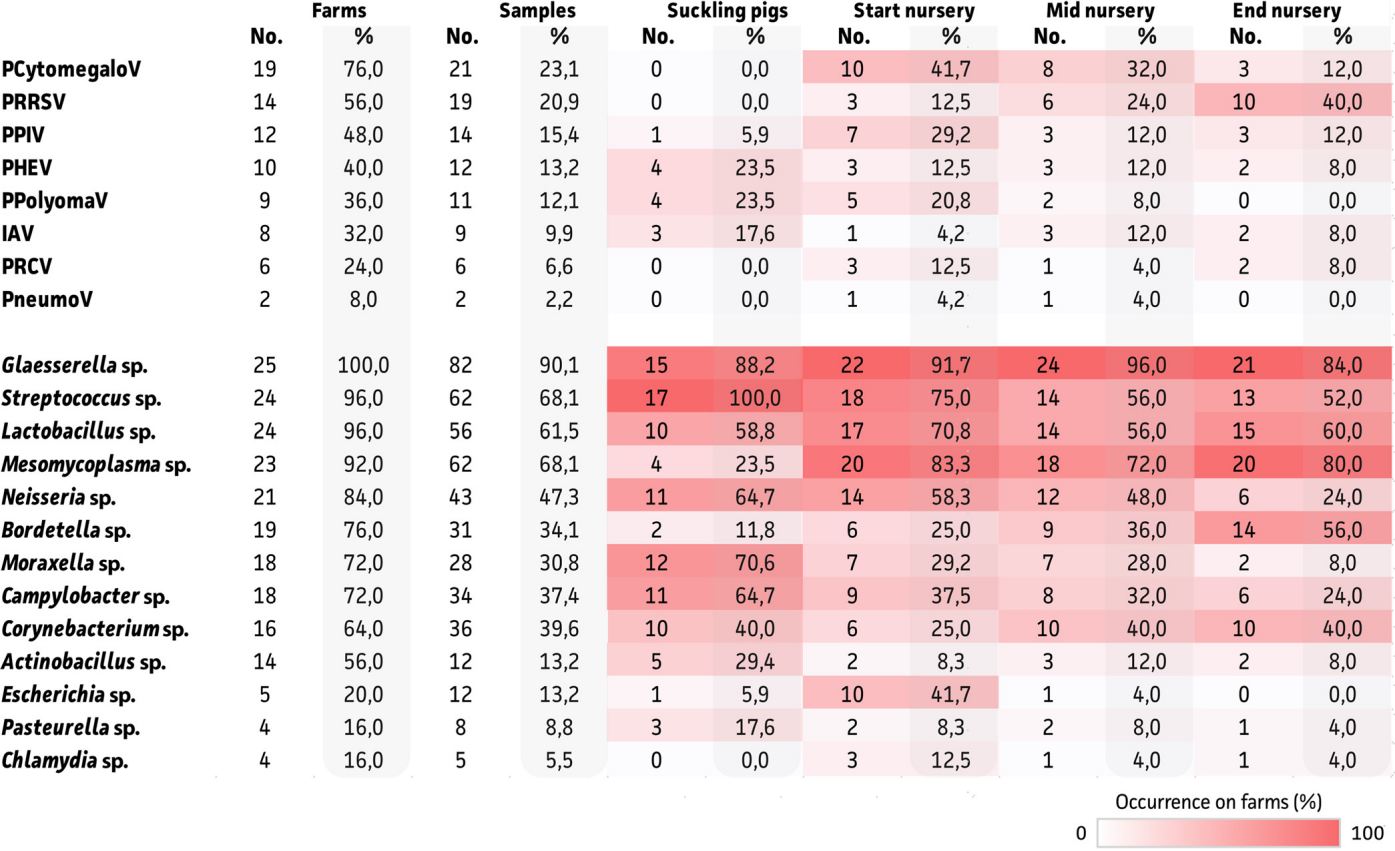

a Figures are based on the farm (*n* = 25) and sample levels (*n* = 91), along with age-dependent stratification. The latter were color-coded showing high (red) and low (white) overall detection rates per age group and indicated as occurrence on farms (%). The viral and bacterial species are ordered according to their overall farm incidence.

### Age-dependent microbial profiles in swIAV endemic infected herds.

As represented in Fig. 2, a clear age-dependent microbial composition could be observed. The suckling piglets showed a more distinct community, compared to the within nursery samples. Overall, an increase in respiratory-associated viruses (Fig. 2A) and a decrease in bacteria (Fig. 2B) were observed across the four age groups. The swIAV detection showed to be age-dependent, showing highest levels in the suckling piglets for both NS and TBS RT-qPCR viral loads. Still, swIAV detection in the NS and TBS samples showed a reverse relationship, as a decrease in the swIAV RT-qPCR viral loads of the TBS samples that were taken from mid-nursery pigs was observed. The respiratory viruses of suckling piglets was represented by five viruses, including PHEV, PIV, PPolyomaV, swIAV, and PCytomegaloV. The bacterial respiratory counterpart was majorly represented by *Glaesserella*, Streptococcus, *Moraxella*, and *Neisseria* species, among others. For viruses, a clear transition from the suckling piglets to the end of nursery could be observed, and this showed a reduction in PHEV and PPolyomaV, the appearance of PRRSV and PRCV, and an increase of PCytomegaloV. Also, a clear shift in community could be observed for the bacteria, which highlighted the presence of *Mesomycoplasma* and *Bordetella* species. Decreases in Streptococcus, *Glaesserella*, *Moraxella*, Escherichia, and *Neisseria* species were also observed. This same transition could be seen in the beta diversity analysis (Bray-Curtis) (Fig. 2C), in which the microbial shifts resulted in a stepwise, left-handed movement of the sample-associated ellipsoids over time (arrow, Fig. 2C). As in the relative abundance plots, the mid-nursery and end nursery pigs represented more similar microbial profiles. Based on a PERMANOVA analysis, PRRSV as well as *Mesomycoplasma*, *Glaesserella*, *Bordetella*, and Streptococcus species were statistically significant contributors to the observed differences between the four different age groups. Next to respiratory pathogens, some other viruses (porcine picobirna-, astro-, parvo-, and rotavirus) and bacteria (Bergeyella and Blautia species) also showed significant contributions (Fig. S1). Indeed, a clear reduction in picobirnavirus and an increase in astrovirus were observed when transitioning from the suckling age group to the start of nursery age group. Rotavirus A is present at a substantial abundance (14% of all viruses) up to the start of the nursery. However, this reduces drastically (3%) from mid-nursery onwards. Even though Fig. S1 appeared to show that some other bacterial species were different across age groups, these results did not reach statistical significance in the beta diversity analysis. As an important note, even though the *Blautia* species were considered to be statistically significant, they were only identified in 3 farms (Table 1).

### Coinfections and swIAV detection.

**FIG 3:**
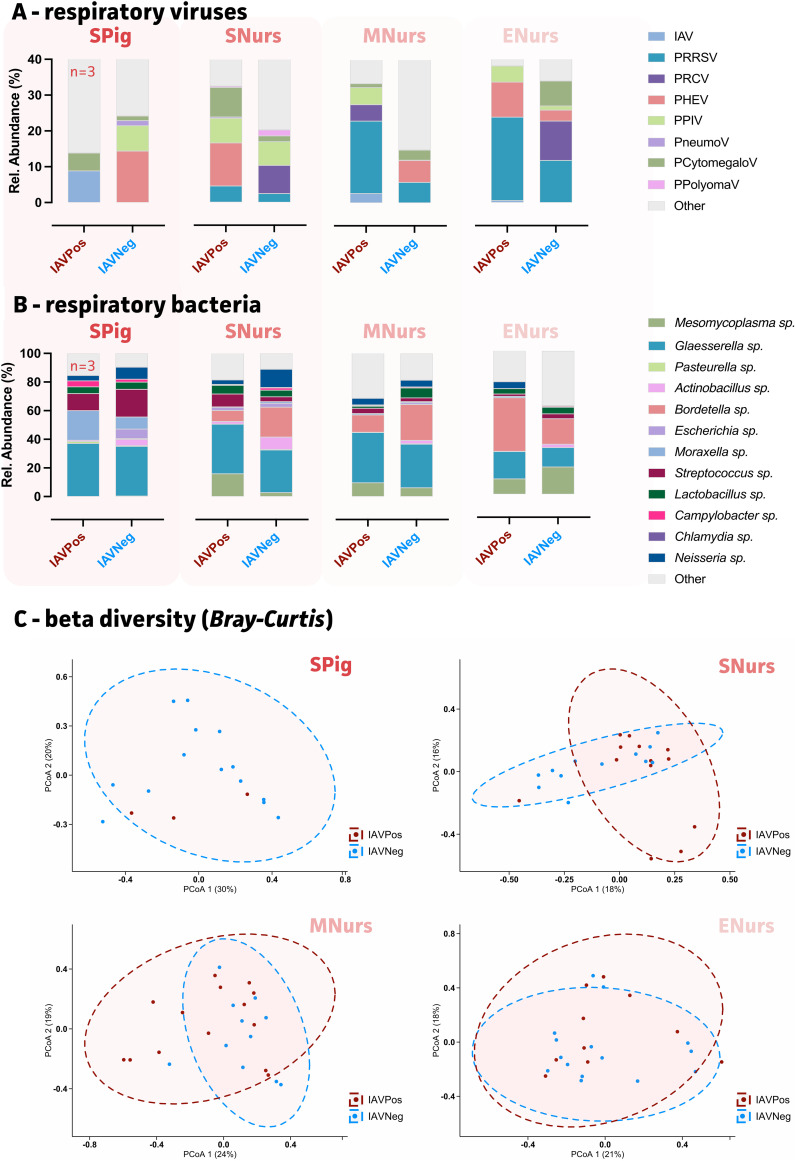
Impact of swIAV presence on the mean relative abundances of relevant respiratory viruses and bacteria in pigs, stratified by age, in 25 farms in Germany. (A) Mean relative abundances (%) of relevant respiratory viruses across age groups. (B) Mean relative abundances (%) of relevant respiratory bacteria across age groups. (C) Principal coordinate analyses (PCoA) of the four age groups for all microbial agents, with IAV-positive and IAV-negative populations represented in red and blue, respectively. Spig, suckling piglets; SNurs, start nursery; MNurs, mid-nursery; ENurs, end nursery.

Coinfections were evaluated with respect to swIAV detection for each age group over the 25 studied farms. As shown in Fig. 3, clear differences in viral and bacterial mean relative abundances could be observed. In suckling piglets, picobirnavirus, astrovirus, and APPV were significantly different between the two populations. For bacteria, *Glaesserella* and *Rothia* species were shown to be significant bacterial genera in swIAV positive farms (Fig. 3; Table 2). An increase in *Glaesserella* and a decrease in *Rothia* species abundances were observed in the swIAV positive group. Whereas PHEV was not associated with the occurrence of swIAV in suckling piglets, at the start of the nursing period, PHEV and PPIV were significant contributors to the swIAV-positive group (Fig. 3A). The swIAV-positive population was characterized by increased abundances of *Glaesserella* sp. and *Mesomycoplasma* sp., along with a reduction in *Bordetella* sp. (Fig. 3B). These big differences were also observed in the PCoA plot, in which both ellipsoids were placed perpendicularly to each other (Fig. 3C). *Glaesserella*, *Bordetella*, and *Mesomycoplasma* species remained significantly increased in the swIAV-positive farms up to the mid-nursery. As shown in Fig. 3A, PRRSV is detected more frequently at that time point in swIAV-positive groups, and it remains elevated up to the end of the nursery. The same was shown for *Glaesserella* species, although a significant reduction in *Bordetella*, *Lactobacillus*, and Streptococcus species characterized the end of the nursery. Also, in the mid-nursery period, picobirnavirues, astroviruses, enteroviruses, and *Coprococcus*, *Parabulkholderia*, and *Faecalibacterium* species were significant contributors. At the end of the nursery, only picobirnavirus, *Faecalibacterium* species, *Prevotella* species, and *Corynebacterium* species were decreased in the swIAV positive group (Table 2). Even though some viral (e.g., PRCV) and bacterial (e.g., *Neisseria* species) pathogens tended to show apparent differences in mean relative abundances, no statistically significant results could be obtained in the beta diversity and PERMANOVA analyses (Table S3).

**TABLE 2:** Significant contributors from PERMANOVA analyses^*a*^

Agent	Suckling piglets	Start nursery	Mid nursery	End nursery
PERMANOVA swIAV positivity^*b*^				
*Glaesserella* sp.	0.001	0.001	0.001	
*Bordetella* sp.		0.001	0.001	0.001
*Mesomycoplasma* sp.		0.002	0.021	0.001
PRRSV			0.001	0.001
PHEV		0.005		
PPIV		0.038		
*Lactobacillus* sp.				0.004
Streptococcus sp.				0.031
*Corynebacterium* sp.				0.049
PERMANOVA clinical signs^*c*^				
*Glaesserella* sp.	0.001	0.001	0.001	
*Bordetella* sp.		0.001	0.001	
*Mesomycoplasma* sp.		0.001	0.035	
PRRSV			0.001	
PHEV		0.006		
PPIV		0.039		
PPolyomaV	0.045			

a For the end of nursery, too few data points were available for group 0 (*n* = 2) and group 1 (*n* = 1). Statistical significance is reported via *P* values. Empty cells represent non-significant measures (ns) for that organism in its respective category.

b Microbes significantly (*P* < 0.05) associated with swIAV presence across the 4 age groups.

c Microbes significantly associated with clinical signs (group 0, group 1, and group 2) across 3 age groups.

### Coinfections and clinical signs (sneezing and coughing index).

**FIG 4:**
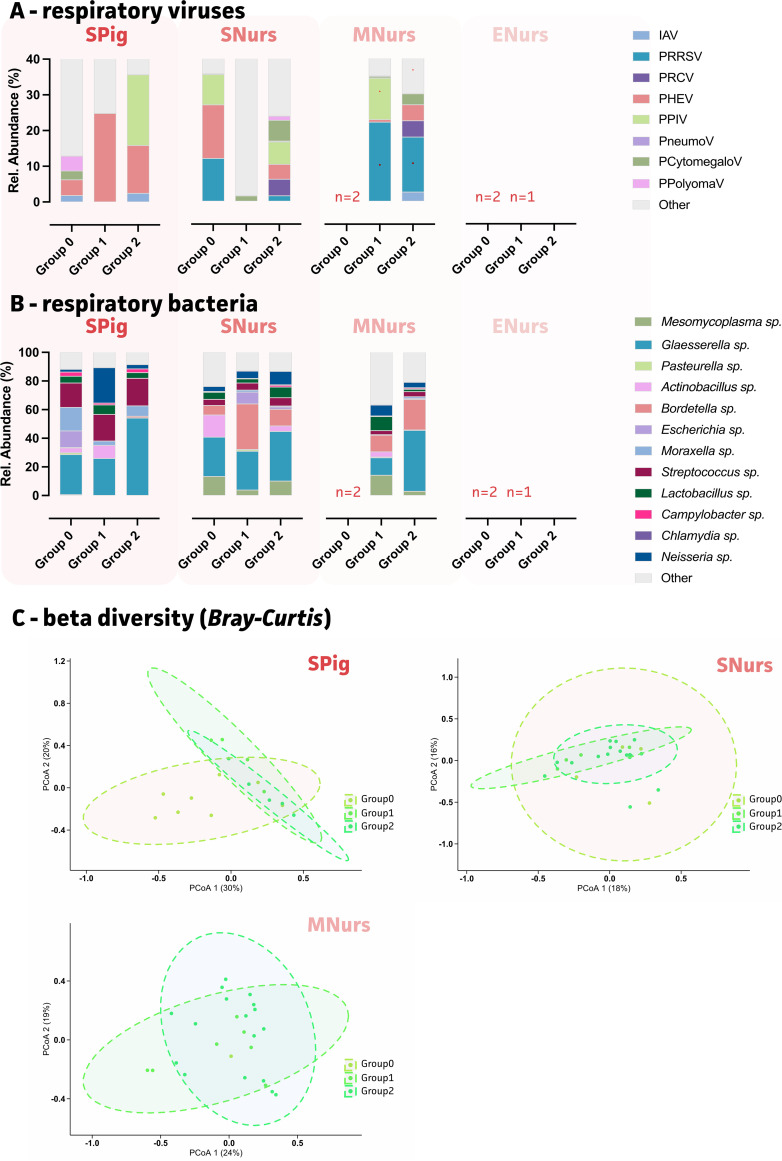
Impact of relevant respiratory viruses and bacteria (mean relative abundances) on clinical signs (coughing/sneezing indices), over ages, for the 25 German farms. (A) Mean relative abundances (%) of relevant respiratory viruses across age groups. (B) Mean relative abundances (%) of relevant respiratory bacteria across age groups. (C) Principal coordinate analyses (PCoA) of age groups for all microbial agents with group 0 (no symptoms), group 1 (elevated coughing or sneezing indices), and group 2 (elevated coughing and sneezing indices) represented in shades of green. For the end of nursery, too few data points were available for group 0 (*n* = 2) and group 1 (*n* = 1). Statistical significance is reported via *P* values. Spig, suckling piglets; SNurs, start nursery; MNurs, mid nursery; ENurs, end nursery.

To determine the relation between coinfections and the observed sneezing and coughing indices, three groups were included in the beta-diversity (Bray-Curtis) and PERMANOVA analyses per age group. These groups included pigs with low indices for both sneezing and coughing (group 0), pigs with either one or both elevated indices (sneezing or coughing [group 1]), and pigs showing increased indices for both sneezing and coughing (group 2). This division allowed for the determination of the viral and bacterial contributors that were associated with clinical signs (Fig. 4). Of note, for the end of nursery group, insufficient samples of group 0 (no respiratory signs) were present, and this precluded proper analyses. In the suckling piglets showing no clinical signs (group 0), porcine polyomavirus (PPolyomaV) and PHEV showed the highest relative abundances (group 0) (Fig. 4A). Only *Glaesserella* species showed a significant increase in group 2 (both coughing and sneezing) (Fig. 4B). As highlighted in the beta diversity (Bray-Curtis) analysis, the suckling piglets without clinical signs (group 0) showed an overall microbial community with a different composition, compared to both group 1 and group 2, for which the ellipsoids nearly collocated. This suggested more similar microbial communities for groups 1 and 2 (Fig. 4C). Picobirnavirus, astroirus, APPV, and *Rothia* species were significant contributors to the observed differences between piglets with and without clinical signs. At the start of nursery, both PHEV and PPIV showed a significant contribution to the differences that were observed across the three groups (Fig. 4A). Interestingly, PHEV was only observed in group 0, whereas PPIV was detected in both group 0 and group 2. Whereas group 1 showed elevated levels of *Bordetella* sp., group 2 was characterized by an increase of *Glaesserella* species and not *Bordetella* species Even though *Mesomycoplasma* species were also significant contributors to the population differences, both group 0 and group 2 showed higher levels, compared to group 1 (Fig. 4B). Finally, in the middle of the nursery, PRRSV was prevalent in both group 1 and group 2, along with high levels of *Glaesserella* and *Bordetella* species, compared to group 1. Too few data points were available for group 0 at this age.

## DISCUSSION

This work showed the potential of third-generation nanopore sequencing in the detection of respiratory pathogens, including the PRDC-associated pathogens, in swIAV endemic infected herds. The *ad random* amplification of the metagenomic workflow principally allowed for the study of any microbe without prior specific pathogen selection. Furthermore, it enabled us to identify other and potentially new viral and bacterial swIAV coinfecting agents within the TBS samples that were taken from the 25 studied German farms. Even though the number of swIAV-positive TBS samples was low, compared to the NS samples, a simple linear regression showed mediocre support for the semi-quantitative nature of the metagenomic workflow. Whereas RT-qPCR approaches are the most sensitive in detecting swIAV, as they only target the influenza virus genome itself, the use of random primers within a metagenomic workflow targets both viruses and bacteria. Hence, having a complex community of both viruses and bacteria represents a “loss” of resolution for samples with lowered swIAV loads. Indeed, our data suggest that swIAV detection in the metagenomics workflow was more successful for samples showing higher TBS RT-qPCR viral loads (778 ± 750 genome copy equivalents per 0.1 mL), compared to those that revealed lower loads (66 ± 60 genome copy equivalents per 0.1 mL) and remained negative in the metagenomic workflow. Hence, swIAV detection in metagenomics indicates positive samples with “high” viral loads, according to the World Organization of Animal Health (WOAH), which is thought to represent the acute stage of an on-going swIAV infection (26). Due to the short swIAV viral shedding of individual pigs (<7 days), several age groups should be sampled, even in epidemic situations, and especially on the farm level. Thus, a negative result using metagenomics does not exclude a previous swIAV infection and a potential role within the observed respiratory disease (27, 28). Still, general care should be taken with Cq values over 36 (<18 genome copy equivalents per 0.1 mL, in our study), as they are difficult to interpret. If the aim is to monitor, screen, or study swIAV in endemic scenarios, RT-qPCR approaches will deliver the highest sensitivity (26, 29). Moreover, the (semi-)quantitative nature of metagenomics has previously been demonstrated for swIAV quantification (30, 31). Even though our work showed a mediocre correlation (R squared of 0.51), this might be in part explained by the overall lowered swIAV viral loads in our data set, as our study focused on swIAV endemic infected herds. The metagenomic workflow that was applied in this study was also extensively validated for the (semi-)quantitative detection of porcine enteric viruses and Mycoplasma bovis. The latter was done in the context of the bovine respiratory disease complex (BRDC), for which an impeccable correlation (R squared of 0.87) was obtained (22, 23). This underlines that, especially in acute stages of infection, metagenomics are a valuable tool with which to identify swIAV, whereas in situations in which the status of the infection is unclear or for monitoring purposes, the detection of influenza via RT-PCR has proven to be the more sensitive method.

Our metagenomic data allowed for the assessment of the overall distribution of respiratory disease-associated coinfections in swIAV endemically infected herds. All of our herds were shown to be swIAV-positive and had a history of respiratory distress. Furthermore, the swIAV viral loads were the highest at the nursery stage, suggesting that swIAV is present at an early age. This was confirmed in recent studies in Europe (16, 28). Hence, swIAV might significantly impact the further clinical course of other pathogens (10, 32, 33). We observed the circulation of PRRSV (56%) as well as *Glaesserella* (100%), Streptococcus (96%), *Mesomycoplasma* (96%), *Bordetella* (76%), *Actinobacillus* (56%), and *Pasteurella* species (16%). An important drawback of the current study is the limited sample size per farm (1 pool per age group), resulting in a total of 17 samples for the suckling piglets and 25 samples per age group (*n* = 3) of the nursery pigs. This resulted in only three swIAV positive samples from suckling piglets and lowered the detection of some pathogens (e.g., PRCV in 6 out of 25 farms). Nevertheless, previous studies on Belgian, Dutch, and Danish farms showed comparable prevalence data for PRRSV (51 to 53%), Streptococcus species (99%), and *Pasteurella* species (15%). For *Mesomycoplasma*, *Bordetella*, and *Actinobacillus* species, our study showed higher levels, compared to previous trials (34, 35). This can be explained by the decision to stick to genus-level reporting within our study. The use of nanopore sequencing represents a slight reduction in raw read accuracy (approximately 97% with R9.4.1 flow cells and the latest base calling models), and this is considered to be a major drawback of the technology (36). Hence, species-level reporting is only facilitated if sufficient reads (usually over 50, covering the complete 16S rRNA gene) are available per species. Even though this was the case for various species across samples, genus-level resolution was opted for so as to facilitate read classifications up to the same taxonomic level. This is thought to be a limitation of our study, although the use of the new R10.4.1 flow cell (raw read accuracy > 99%) will contribute to single-read species-level classification in the future (37). Interestingly, PCV-2 was not detected in any of our samples. This is surprising, as PCV-2 is considered to be an important player in porcine respiratory disease. Even though PCV-2 and swIAV coinfections are frequently observed (38, 39), data on dual infection challenges are scarce (40). Recent studies showed low PCV-2 levels (10 to 20% [34, 35]) in respiratory samples. Interestingly, the study of Goecke and colleagues (2020) showed the presence of PCV-2 in only a few samples without a clear correlation of clinical signs (35, 40). This is important, as it questions the actual role and presence of infectious virus within lower respiratory tract samples. Various reports on high levels of PCV-2 within the respiratory tract might be results of residual nucleic acids rather than life infectious virus (3, 41–43). This is supported by the fact that PCV-2 is known to be cell-associated (e.g., lymphoid tissue), and, thus, the current sampling strategy might represent the detection of non-infectious PCV-2 material, such as its nucleic acids (44). The latter is important, as the metagenomic workflow includes an enzymatic depletion of free-floating host nucleic acids, which will also remove residual PCV-2-associated nucleic acids when not protected by a viral capsid (45). Similar results were obtained for serum samples, in which a clear cutoff (approximately 10^7^ genome copies) was seen for the detection of PCV-2 in relation to the qPCR Cq values (unpublished data). Still, swIAV and PCV-2 coinfections are common, but they were not shown to influence virus replication or worsen clinical symptoms (10, 40). However, this does not imply that PCV-2 infections do not contribute to respiratory disease and/or PRDC. In addition, its identification is thought to be age-dependent, as an increase from 11% (6- to 11-week-old pigs) to 27% (12- to 25-week-old pigs) was observed in Belgian TBS samples (16). To ensure its relevance, lymph nodes and/or lung tissues of pigs should be collected to determine PCV-2 infections via immunohistochemistry and qPCR. Whereas the aforementioned pathogens are considered core pathogens within PRDC, PCytomegaloV was also detected in 76% of all farms, which is in line with previous reports of nursery and fattening pigs combined (96%) (35). Next to these viruses, our study also shed light on the presence of PPIV (48%), PHEV (40%), PPolyomaV (36%), and PRCV (24%) in swIAV-infected German farms. To the authors’ knowledge, this is the first time that the circulation of PPolyomaV in swine is shown, as only a limited number of reports have been published in recent years (46). Even though most samples originated from pigs with respiratory diseases, its clinical relevance is not yet known (46). To completion, various microbes with enteric tropism were also identified in the present study. These included astro-, picobirna-, entero-, and rotaviruses, along with various bacteria (*Prevotella*, *Faecalibacteirum*, *Rothia*, *Corynebacterium*, *Bergeyella*, *Parabulkholderia*, *Coprococcus*, and *Blautia* species). Most of these are thought to be sampling contaminants, as the TBS sampling procedures might result in contact with mucus and tonsils, which harbor a wide variety of environmental microbes. Indeed, most of these microbes have been identified with the pig gut microbiome (47–51). Interestingly, some of these have been associated with respiratory disease, as exemplified for porcine astrovirus type 4 and *Corynebacterium*, which is a known opportunistic pathogen that causes purulent infections (e.g., Corynebacterium pyogenes) (52, 53). The latter is also considered to be important from the One Health perspective, as different cases of human infections after contact with pigs have been reported (54). Hence, this highlights the potential added value of metagenomics-driven diagnostics for both animals and humans. Nevertheless, to deliver useful and relevant diagnostic reports to veterinary practitioners, analytical and interpretative expertise will be required. This includes the extensive validation of any detected microbes against databases (e.g., NCBI) and scientific literature. Also, veterinary practitioners should properly evaluate which sample to use so as to tailor treatment and/or management changes on a farm. Even though oral fluids have become widely used for diagnostics, their use in metagenomics is questionable. While they potentially deliver information on relevant pathogenic microbes, they also detect a jungle of irrelevant environmental microbes, making final interpretation an even bigger challenge (41, 48, 55). Veterinary practitioners most often rely on antigen/antibody-based tests, as they are the cheapest (i.e., 10 to 20 EUR per sample [14, 56]). However, paired sera are required to distinguish past infections from ongoing infections, and this impacts their turnaround time (i.e., 3 weeks [14]). Whereas these tests represent indirect detection methods, bacterial cultures and molecular tests (e.g., PCR) are also applied. While bacterial cultures have a cost of approximately 30 EUR per sample, they are prone to contamination, require viable material, and are not applicable for all bacteria (e.g., *Mycoplasma* sp. [22, 56, 57]). Alternatively, molecular methods allow for the identification of inactivated and viable material in a wide variety of samples. Still, multiplex PCR approaches that simultaneously target multiple pathogens are scarce, do not target all pathogens, are not routinely applied, and are offered at a high cost of approximately 125 EUR (16, 56). For the same cost, a complete metagenomic screening that includes the high-resolution detection of both viruses and bacteria, without the need of prior pathogen selection in the context of acute infections, can be performed. Furthermore, in addition to the identification of viruses and/or bacteria within a sample, routine diagnostics often still require additional virotyping and antimicrobial susceptibility testing, which increase the total cost of the diagnostics and the turnaround time. Additionally, metagenomics is thought to be in favor, upon its future development and fine-tuning (58, 59).

**FIG 5:**
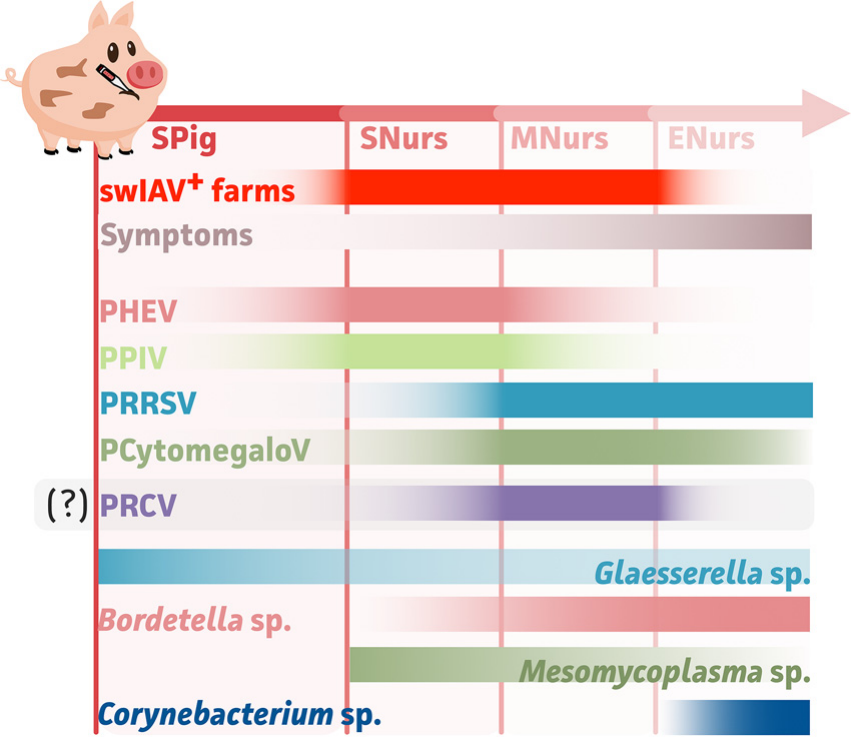
Schematic overview of the dynamic interplay of various microbes in PRDC. This schematic representation shows the hypothesized (and significant) contributors to swIAV occurrence and the clinical signs, as determined from 25 swIAV suspicious German farms. Solid zones represent the most important contribution at the indicated age group. Faded zones represent the reduced presence and/or importance of the indicated microbes. For PRCV, no significance was observed, although it showed an apparent important role in clinical manifestations in the start and mid nursery periods. Spig, suckling piglets; SNurs, start nursery; MNurs, mid nursery; ENurs, end nursery.

In addition to overall pathogen detection, our data allowed us to address the dynamics of respiratory agents, as respiratory microbial communities were shown to be highly age-dependent. This result is not surprising; as is the case for the gut microbiome, significant differences have been observed throughout the different life stages of pigs (49, 51). To the authors’ knowledge, this is the first study to describe multiple viral and bacterial respiratory agents in the context of swIAV infections within a single assay. Thus, a general hypothesis could be drawn, regarding which pathogens to consider on swIAV endemic infected herds with respiratory signs at each age stage (suckling up to late nursery), as is summarized in Fig. 5. This was done using a beta diversity analysis on the sample-wide microbial abundances, which allowed for the assessment of microbes with significant contributions to the observed microbial community differences. The Bray-Curtis index provides a well-defined concept with which to study ecological dissimilarities and summarize multidimensional data (e.g., multiple microbes over various samples) in a simple 2D space via ordination analyses (60). With this method, it was possible to determine the microbes with the highest impact in the context of swIAV detection and clinical signs (coughing and/or sneezing indices). The least impact of respiratory pathogens was seen at the suckling piglet stage (2- to 3-week-old pigs), as the lowest numbers of pathogenic respiratory viruses and bacteria were identified here. Also, the lowest coughing and sneezing indices were observed in this population. This might be explained by the maternal immunity that delivers protecting antibodies and hence prevents clinical manifestations (61). Still, apparently higher (10- to 20-fold) levels of PHEV and PPIV were found in suckling piglets with clinical signs, although they were not considered to be statistically significant. More important, a significant impact of *Glaesserella* species was seen on the suckling piglets if sneezing and coughing were observed. This might be due to its secondary/opportunistic nature if a viral agent (e.g., PHEV or PPIV) was present (62). It is only at the start of the nursery that both PHEV and PPIV were shown to be coexisting in swIAV-positive populations. Further, to the authors’ knowledge, our study shows the first evidence of swIAV coinfections with PHEV and/or PPIV. Conversely, they were not associated with increased clinical signs. So far, few diagnostic tools for PHEV have been developed; thus, metagenomics represents an easy way to determine the presence of PHEV within a herd (63). PHEV is known as a causative agent of vomiting and wasting disease (VWD) or encephalomyelitis, and it is mainly seen in piglets below 4 weeks of age. It is the only swine-infecting coronavirus that is known to exhibit neurotropism. Even though various clinical manifestations have been reported over time, it is considered endemic in most swine herds worldwide, due to its subclinical circulation (61, 64). Still, it is regarded as an important pathogen in farms with high gilt replacement rates if animals have not been previously exposed to PHEV. These naive gilts do not deliver protection to their offspring via lactogenic immunity (65). Whereas PHEV was first isolated in 1962, porcine parainfluenza virus type 1 (formerly known as porcine respirovirus type 1) was detected more recently in 2013 in Hong Kong (66–68). While the inoculation of pigs resulted in high levels of replication and shedding, no to mild clinical symptoms (e.g., small lesions in the lungs) have been associated with a PPIV infection (69). Moreover, the virus was shown to be commonly circulating in different farms across the world (66–68, 70–73).

In our study, *Glaesserella* species remained an important bacterium at the start of the nursery phase (4- to 6-week-old pigs), although *Mesomycoplasma* and *Bordetella* species also became predominant contributors. With a focus on *in vivo* trials, swIAV was not shown to impact the colonization and proliferation of either G. parasuis or B. bronchiseptica. Still, swIAV coinfections with B. bronchiseptica showed enhanced clinical signs in coinfected animals (74) as well as elevated swIAV nasal shedding (75). Another study showed a delay in swIAV clearance in the presence of B. bronchiseptica (76). In our study, we observed an age-dependent decrease and increase of swIAV and B. bronchiseptica, respectively. This age-dependent swIAV decrease was also observed in a recent Belgian study that did not include data on B. bronchiseptica (16). The increase of B. bronchiseptica over time is likely because of its opportunistic/secondary nature in respiratory diseases in older pigs (77). Whereas *Bordetella* species became more important by the end of the nursery phase, a reduction and increase of the *Glaesserella* and *Mesomycoplasma* species populations, respectively, were observed from the start to the end of the nursery period. This is interesting, as coinfections of swIAV and Mesomycoplasma hyopneumoniae were shown to result in an earlier influx of CD163^+^ cells and neutrophils (42, 78). The CD163 receptor is known to be the main receptor for PRRSV, of which the relative abundance also increased from the start of the nursery onwards (79). Overall, this is associated with increased sneezing and coughing indices (worse clinical signs) at the start of the nursery, which was shown to be associated with the significant contribution of PRRSV. Unfortunately, our data did not allow for the drawing of proper conclusions on the clinical signs for the mid-nursery and end of nursery age groups, as only a few pigs showed no clinical signs. Nevertheless, it has been shown on various occasions that coinfections with PRRSV can result in a synergism that can result in a worse clinical outcome (6, 80–83). Indeed, PRRSV was most abundant in swIAV positive populations, in which coinfections with *Mesomycoplasma*, *Glaesserella*, *Bordetella* species or PHEV, PPIV, or PRCV were often observed. Dual infections with swIAV and PRRSV have been widely studied. These studies highlighted the biggest impact on disease outcome, although discrepancies were observed between various studies (10). While most studies showed no impact on swIAV shedding (84–87), a PRRSV infection reduced swIAV vaccination efficacy (84), swIAV delayed the PRRSV infection (6), and a lower PRRSV viral load was observed in bronchoalveolar lavages from pigs that were coinfected with swIAV, compared to a PRRSV mono-infection (88). When considering bacterium-virus interactions or *vice versa*, M. hyopneumoniae increased PRRSV replication in the lymphoid tissue and blood (89, 90), and a PRRSV infection resulted in increased levels of G. parasuis and Mesomycoplasma hyorhinis (91). The latter should not be forgotten, as it is often coisolated with M. hyopneumoniae from pneumonia-like lesions. The same is true for Mesomycoplasma flocculare. They are thought to play a role in immunomodulation within the lung environment (92). Still, to date, little scientific evidence is available on these rarely studied species. Our data showed the increased detection of *Mesomycoplasma* species, and this was followed by increased viral loads of PRRSV. Interestingly, PCytomegaloV and PRCV were also present at a higher relative abundance from the start of the nursery. The former virus is known to be immunosuppressive and able to alter the immune responses in T cells and macrophages, which thereby promotes respiratory diseases (e.g., PRRSV) (93). For PRCV, most infections progress in a subclinical way. The virus has been widely studied as a pathogen in porcine respiratory disease, showing high PRCV replication in the lung, but its impact and disease outcome are thought to be highly dependent on the strain (94). The detection of PRCV from the start of the nursery onwards is in line with the results of previous reports that suggest an endemic infection around 5 to 8 weeks (1, 6, 16). Even though PRCV seems to be present only in pigs showing both sneezing and coughing symptoms, no significant contribution was observed. Hence, its exact role within the respiratory disease and PRDC still remains to be elucidated further (1). A recent study by Martin-Valls and colleagues (2022) showed that PRCV and PCytomegaloV were more frequently isolated from swIAV-positive farms (11). However, their results were based on samples that were collected from nasal swabs and, as such, might not be able to be extrapolated to the deeper respiratory tract (i.e., TBS sampling). Also, for Streptococcus species, no statistically significant association with either swIAV or clinical signs in any of the studied age groups could be made. This is in line with speculation on the effective role of S. suis within porcine respiratory disease, as it is known to be a common inhabitant of the respiratory tract. So far, few *in vivo* dual infections have been performed, showing no impact of swIAV on bacterial colonization and proliferation. Still, higher swIAV viral loads were identified in the nose and lungs in S. suis coinfected pigs, as were more severe clinical signs and pathological lung lesions (95). Coinfections with PRRSV showed higher pathogenicity and mortality (82, 96). It is important to note that S. suis can be divided into up to 35 serotypes, of which serotype 2 has been most often associated with disease (10). Hence, it is thought that the environment (as the third part in the respiratory disease triangle) contributes to outbreaks and coinfections only if virulent strains are circulating (62). Still, *in vitro* experiments showed that highly virulent swIAV promoted the adherence, colonization, and invasion of S. suis (8).

In conclusion, we showed the added value of a third-generation nanopore sequencing diagnostics workflow in the in-depth understanding of the porcine respiratory disease. Compared to the often costly diagnostic procedures that are applied for PRDC diagnostics nowadays, here, we presented a cost-efficient and informative tool (2, 3, 34, 63). Its wider implementation for veterinary practitioners will require proper actionable reporting, which should only include relevant pathogens. To filter out contaminating and irrelevant microbes, the dynamics of the respiratory disease should be considered. Moreover, our data (from 25 endemically swIAV-infected German farms) allowed us to address (and filter out) the most important pathogens to be used in future diagnostics. In addition, our data suggest that more efforts should be put into investigating coinfections and lesser-known pathogens (e.g., PHEV and PPIV) as well as their potential roles in porcine respiratory disease.

## MATERIALS AND METHODS

### Study design and animals.

This cross-sectional study was conducted in 25 prospectively selected swine farms from all regions of Germany (Fig. 1A). All farms were sow farms with attached nursery units that were suspected to have an endemic swIAV infection due to a history of swIAV infections. These farms had tested positive for swIAV before the initiation of the study. Based on information from the farmers, respiratory distress was frequently observed in all farms. Both nasal and TBS samples were collected between March of 2021 and February of 2022. On every farm, five nursery pigs were sampled from three different age groups, representing the start (4 to 6 weeks of age), mid (7 to 8 weeks of age) and end (9 to 10 weeks of age) of the nursery period. From farm 8 onward, nasal and TBS samples were also collected from four suckling piglets (2 to 3 weeks of age) that originated from four different litters of gilts. The sampling protocol and procedures were approved by the Ethics Commission of the Ludwig Maximilians-Universiteit (LMU) Munich (accession number PRJEB59352).

### Sampling procedures.

Nasal swabs (NS) were collected using rayon swabs (Dryswab, catalog number MW112/MW113, Check Diagnostics GmbH, Westerau, Germany) and were immediately placed in Virocult (Check Diagnostics GmbH, Westerau, Germany). The TBS samples were collected as described previously (12, 13). Briefly, piglets were either fixed beneath the arm of an assisting person or restrained by using a snare, corresponding to the sizes and weights of the animals. A mouth gag was placed between the upper and lower jaw, and during inspiration, a sterile catheter (DCT-Nelaton Katheter CH10/CH12; servoprax GmbH, Germany) was inserted into the trachea. When coughing was provoked, it was assumed that the bifurcation tracheae was reached, and the sampling was considered successful. The tip of the catheter was cut and transferred into a sterile 50 mL Falcon tube containing 4 mL of sterile phosphate-buffered saline (PBS). Additionally, udder skin wipes, surface samples, and oral fluids were collected on all farms in different age and production groups for the virological detection of swIAV via RT-qPCR (manuscript in preparation) (97). Clinical signs were assessed using a coughing and sneezing index (98). This index is composed of two runs and two independent observation periods. Each run is represented by a 3-1-3 scheme, in which all coughing and sneezing episodes were recorded in the first 3 min. This was followed by a 1 min break, after which episodes were recorded for 3 min again. Each time a new run was initiated and after each break, the pigs were roused (except for the suckling piglets). A minimum of 20 animals per pen (or in 2 adjacent pens) were observed. The second run was performed within the same compartment but with animals within a different pen. Single coughing/sneezing events were recorded if they were separated by at least 10 seconds. The final indexes were calculated as is shown in Equation 1. All of the collected metadata can be found in Table S1.

(1) indexsneezing/coughing=total  events  sneezing  or  coughing total  animals in pen×observation  period  (min.)

### Assessment of swIAV incidence on the farms.

Individual NS and pooled TBS samples were subjected to the detection of swIAV using an RT-qPCR approach. The freshly collected TBS samples were pooled by age level in an even ratio after submission to the PathoSense laboratory (Merelbeke, Belgium). Briefly, in the nursery, three pools per farm (with each pool consisting of five animals in each age group), and in the suckling period, one pool comprised of the four suckling piglets, was investigated. Pooled samples originated from animals of the same barn. A modified generic matrix (M)-gene specific influenza A virus RT-qPCR was performed according to the methods reported by Spackman (2014) (97). In short, nucleic acids were extracted using a NucleoMag VET Kit (Macherey-Nagel GmbH, Dueren, Germany) on a Biosprint 96 System, enabling semi-automated processing. This was followed by RT-qPCR using an AgPath-ID One-step RT-qPCR Kit (Thermo Fisher Scientific, USA) on a CFX96 Touch Real-Time PCR Detection System (Hercules, CA, USA). All of the resulting Cq values were converted to genome copy equivalents per 0.1 mL on the basis of quantified RNA run-off transcripts of a standard. An overview of these results can be found in Table S1.

### *Ad random* viral and bacterial metagenomics using nanopore sequencing.

For the TBS samples, fresh pooled samples (four animals per pool in the suckling piglets and five animals per pool in nursery the pigs) were collected and submitted to the PathoSense laboratory (Merelbeke, Belgium). Transport was done using overnight courier services in polystyrene boxes that were cooled with ice packs (4°C). In general, the samples were purified using a novel patented sampler (patent WO2020260583) to enrich for intact viral and bacterial pathogens before nuclease treatment and nucleic acid extraction. Further preparation of the samples for viral and bacterial metagenomics was done using an in-house developed sample-collection-to-diagnostic-interpretation workflow, as described previously (22, 23, 58, 99). Metagenomic sequencing was done on a GridION X5 (ONT) sequencing device using R9.4.1 flow cells (ONT) in combination with a Rapid Barcoding Kit (SQK-RBK004, ONT). Reads were base called using the “super accurate” base calling model in Guppy (v6.2.7; ONT). As described before, this workflow enables the identification of both DNA/RNA viruses and bacteria in an *ad random* manner via the taxonomical classification of the reads against a curated database. To exclude false positive and aspecific hits, host material was removed using the Sus scrofa reference genome (SusScr11) along with an additional validation against the complete NCBI nucleotide database. The results were reported by PathoSense in a semiquantitative way, as represented by the relative abundances (%). The latter was calculated based on the number of detected reads, compared to a spike-in control virus that was added to each sample prior to the filtration with the sampler. At least two genetic reads were required before a sample was considered to be positive for a given microbe. The relative abundances for viruses and bacteria were calculated separately and can be found in Table S1.

### Linear regression of swIAV sequencing reads.

To evaluate the (semi-)quantitative nature of the detected reads, swIAV RT-qPCR data (genome copy equivalents per 0.1 mL) were used in a simple linear regression with the normalized swIAV relative abundances (%). Even though overall lowered swIAV viral loads were observed, the goodness-of-fit, using the R squared measure along with a 95% confidence interval of the best-fit line, was generated in GraphPad Prism (v9.4.1). All of the other statistical tests were also performed in GraphPad Prism, as is indicated throughout the manuscript.

### Evaluation of cocirculating viral and bacterial agents.

The relative abundances of all of the microbes within each sample were subjected to a beta diversity analysis using the Bray-Curtis dissimilarity index, as calculated using the vegdist tool in vegan (v2.6-2) (100). A principal coordinate analysis (PCoA) was performed using cmdscale (stats v4.2.1) and was visualized using ggplot2 (v3.3.6) (101). A PERMANOVA analysis with 999 permutations was performed using envfit in vegan. Statistical significance was determined based on *P* values being <0.05. Relative abundances below 0.1% were omitted, along with viral and bacterial species that were only detected in three or fewer farms, unless they were known to have respiratory relevance. Our data were divided into two groups: one based on swIAV detection and one based on clinical signs (low sneezing/coughing indices [group 0]; either of one index elevated [group 1]; and both indices elevated [group 2]). Complete outputs can be found in Tables S2, S3, and S4.

### Data availability.

The read files were deposited into the European Nucleotide Archives (ENA) under the project accession number PRJEB59352 (ERS14550157 to ERS14550248).
