# Supplementary figures and images for: Viral and Bacterial Profiles in Endemic Influenza A Virus Infected Swine Herds Using Nanopore Metagenomic Sequencing on Tracheobronchial Swabs
Source: Microbiol Spectr. 2023 Feb 28;11(2):e00098-23. doi: 10.1128/spectrum.00098-23 (PMC10100764; doi:10.1128/spectrum.00098-23)

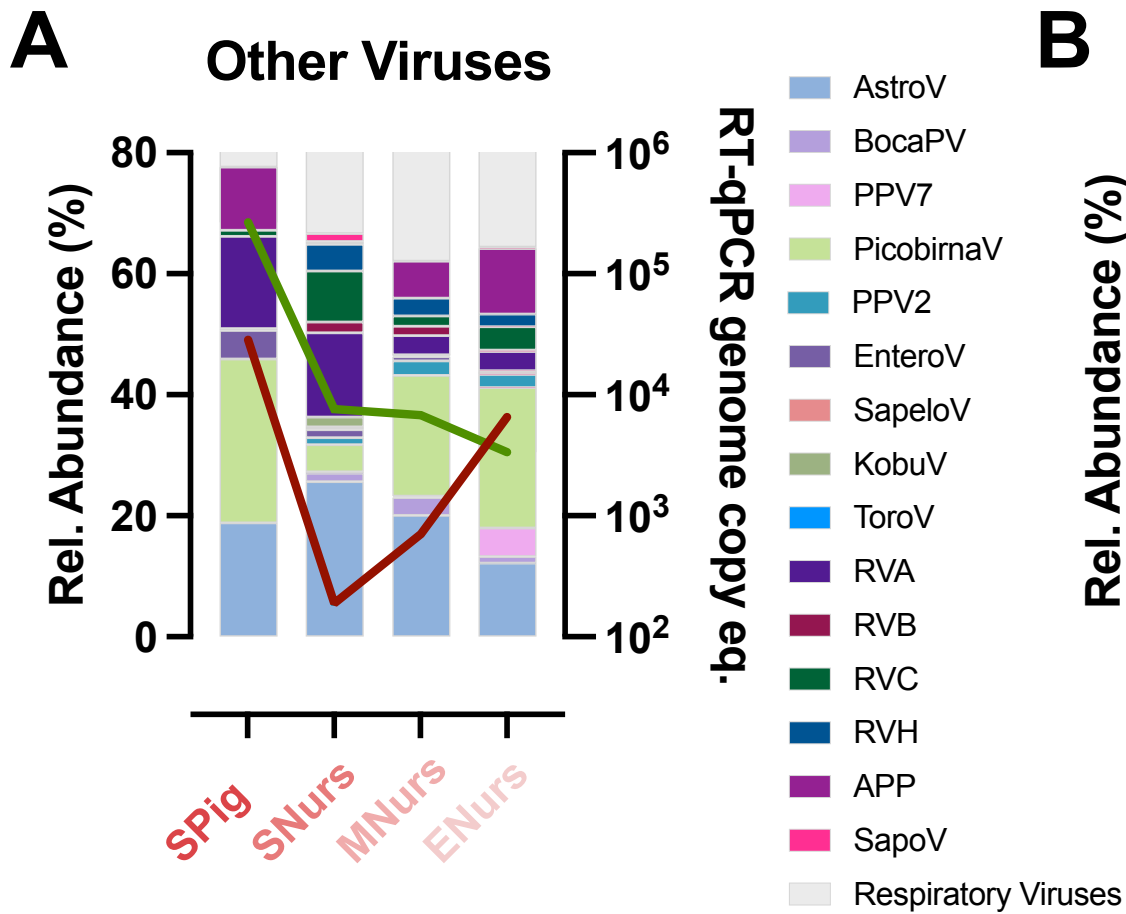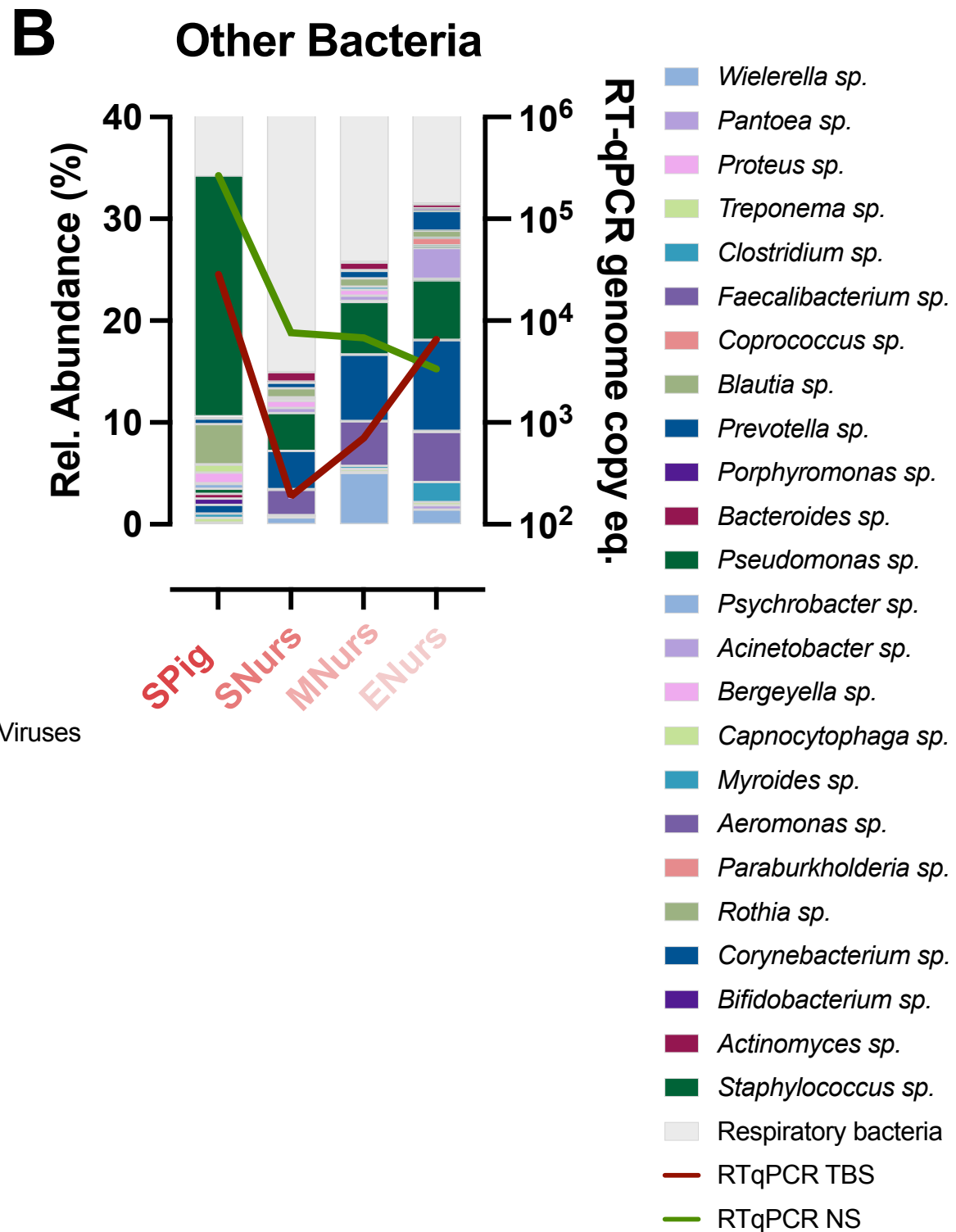

Supplement: Supplemental file 1 — Supplemental material. Download spectrum.00098-23-s0001.pdf, PDF file, 0.06 MB [file spectrum.00098-23-s0001.pdf]
